# The *Saleema* initiative in Sudan to abandon female genital mutilation: Outcomes and dose response effects

**DOI:** 10.1371/journal.pone.0213380

**Published:** 2019-03-12

**Authors:** W. Douglas Evans, Cody Donahue, Jeremy Snider, Nafisa Bedri, Tibyaan A. Elhussein, Samira Ahmed Elamin

**Affiliations:** 1 Milken Institute School of Public Health, The George Washington University, Washington, DC, United States of America; 2 Child Protection Unit, UNICEF, Khartoum, Sudan; 3 Fred Hutchinson Cancer Center, University of Washington, Seattle, WA, United States of America; 4 Gender and Rights Advocacy Center, Ahfad University for Women, Khartoum, Sudan; 5 Independent Research, Khartoum, Sudan; Mälardalen University, SWEDEN

## Abstract

**Purpose:**

The overall goal of the *Saleema* Initiative in Sudan is to promote long-term abandonment of female genital mutilation and cutting (FGM) through a contribution to changing social norms, attitudes, and intentions related to the practice. The initiative aims to create positive cultural associations with a girl remaining uncut, a new social norm. *Saleema* hypothesizes that *branding* the alternative to FGM (abandonment) will promote social norms change. In 2014, the lead author designed a monitoring and evaluation framework for *Saleema* in partnership with UNICEF, the National Council for Child Welfare (NCCW), and local organizations.

**Methods:**

The *Saleema* evaluation aimed to evaluate the effectiveness of the campaign in reducing pro-FGM social norms. A quasi-experimental design controlled for dosage of campaign messages delivered across the 18 states in Sudan to measure a dose-response effect. We operationalized social norms through a 4-item scale validated in previous research.

**Results:**

This paper reports on quantitative evaluation findings based on data gathered in from 2015–2017 and focuses on the dose-response relationship between Saleema exposure and changes in FGM social norms. We found that self-reported exposure was associated with reduced pro-FGM social norms (coeff. = -0.329, *p* < .001). Additionally, higher doses of *Saleema*, measured through an exogenous measure of campaign event exposure from an independent monitoring system was associated with reduced pro-FGM social norms (coeff. = -0.146, *p* < .001).

**Conclusions:**

*Saleema* was effective in reducing pro-FGM social norms. It is a promising strategy and findings contribute to the growing literature on social norms approaches to behavior change.

## Introduction

The World Health Organization (WHO) and other global health and development organizations, including the Department for International Development (DFID) and US Agency for International Development (USAID), note female genital mutilation and cutting (FGM/C, herein FGM) is a widespread and harmful practice [1]. There are 4 main types of FGM, ranging in severity from Type I to IV.^2^ FGM prevalence is highest in 27 countries in Africa and the Middle East [2–3]. In Sudan, FGM is highly prevalent among all age groups of girls and women, with an estimated national prevalence rate of 87% among women aged 15 to 49 years [4]. Recent data from the Multi-Cluster Indicators Survey (MICS) suggest there is a steady, though modest, decline in the rate of FGM among the younger age cohorts (age 25 and below) and 52% of women believe the practice should stop [4].

The social practice of FGM has deep cultural and historical roots and has connotations with religious practice. While there is debate as to its origins and social foundations [5–6], many development organizations such as UNICEF and WHO view FGM as a social norm, or beliefs about how individuals in the community should behave (injunctive norms, beliefs about what should be), and what most people in the community actually do (descriptive norms, empirical beliefs) [7]. Thus, when a social norm such as FGM is in place, families and individuals engage in the practice because they believe that it is expected of them and is prevalent. Without these perceptions, alternative views of FGM would emerge, the social norm would be weakened and practice would be abandoned over time. Changing social norms has been hypothesized as a key step in behavior change [8–9]. The current study tests this hypothesis.

The *Saleema* Initiative is a Sudanese program supported by the government that receives technical and financial assistance from UNICEF and supports the protection of girls from FGM, particularly in the context of efforts to promote collective community abandonment of the practice. The broad objective is to change the way that people talk about FGM by promoting wide usage of new positive terminology to describe the natural bodies of girls and women. Saleema also aims to stimulate new discussions about FGM at family and community levels–new both with regard to who talks to whom (‘talk pathways’) and the specific issues discussed (‘talk content’).

The overall goal of the *Saleema* Initiative is to promote long-term abandonment of FGM through a contribution to changing social norms, attitudes, and intentions related to the practice [10]. The immediate measureable outcome of the program is to increase social acceptance of uncut girls, as reflected in increased use of the positive term *Saleema* and in normative beliefs about the acceptability of being uncut (see Saleema.net) [10].

Social Norms Theory forms a conceptual foundation for the *Saleema* Initiative [10–11]. FGM continues to persist one generation to the next because it is based on social normative beliefs about how individuals in the community should behave (injunctive norms, beliefs about what should be) what most people in the community actually do (descriptive norms, empirical beliefs) [11]. When a social norm such as FGM is in place, families and individuals engage in the practice because they believe that it is expected of them and is prevalent. Creating alternative views is thus a key step in social norm change [12]. When subjective and descriptive norms are in conflict, many people follow descriptive norms, what they perceive the prevalent behavior to be [13]. Even if a norm is considered subjectively important (what should be) if individuals observe non-conformity they will be less likely to follow the norm [13]. Spreading a social norm that modern Sudanese society no longer practices FGM is the long-term goal of *Saleema*.

The *Saleema* initiative, and in turn research to evaluate its effects on FGM social norms and behavior in Sudan, is based on a theoretical model of social norms and the potential to change those norms through creating an alternative narrative and identity for abandonment of the practice. The basis of this model is that pro-FGM social norms may be changed through social dialogue and providing role models showing that uncut girls are socially acceptable. The norm to be changed is that a girl remaining uncut is associated with the word *Saleema*, which connotes remaining whole and as God given. This new norm will become widely accepted within the national culture and represent the growth and progress of Sudanese society [10, 13].

In 2014, the lead author designed a monitoring and evaluation framework for *Saleema* in partnership with UNICEF, the National Council for Child Welfare (NCCW), and other government and non-governmental organizations, including Ahfad University for Women (AUW), Gender and Rights Advocacy Center (GRACe) (hereafter GRACe), which carried out field work for the evaluation. The *Saleema* evaluation had four aims:

1. Measure social norms about FGM over time
2. Measure dosage of *Saleema* delivered and variation in exposure to the initiative across Sudan
3. Determine whether there is a dose-response relationship between higher exposure to *Saleema* and improved normative beliefs about FGM
4. Examine how different populations and regions within Sudan respond to *Saleema* and the relationship between these variations and *Saleema* outcomes

Additionally, the evaluation examined the role of the *Saleema* social marketing and branding strategies in promoting changes in FGM social norms [14]. The brand equity construct–a multi-dimensional scale that measures identification with the *Saleema* brand and the benefits of FGM abandonment that it promotes–is hypothesized to be the mediator of behavior change [15–17].

## Methods

### Intervention

The Saleema campaign was implemented through four main activities across Sudan: 1) Sufara *Saleema* Campaign, 2) *Saleema* Colors Campaign, 3) community dialogue, and 4) Born *Saleema* Project [18]. These activities included publicly pledging to abandon FGM and support the Saleema initiative, wearing Saleema colors as a sign of support, public dialogue on the existence of FGM, its role in society, and the need for abandonment, and pledges not to cut newborn daughters immediately after birth [19].

Each component of the campaign was conducted in a public setting, including large gatherings (e.g., community dialogue), which reached local populations in an effort to change cultural acceptance of uncut girls as a social norm. These activities collectively aimed to increase public commitment, dialogue, and self and collective efficacy to abandon FGM. Details on the intervention activities at the local level are available elsewhere (https://www.unicef.org/infobycountry/sudan_55692.html) [18].

### Design

The study design was a quasi-experiment in which levels of *Saleema* exposure varied by geographic location and over time within Sudan. There are 18 states within the country. From the outset of the study, investigators assumed there would be natural variation in exposure to community-level *Saleema* campaign activities among the regional population. This is due to variable resources and efforts of local *Saleema* partners and variable capability to implement the campaign by location. As a function of higher and lower levels of exposure, we anticipated that outcomes measured would vary.

Prior to these activities, the research team collected pre-test data at wave 1 (W1) in December 2015, followed by waves 2 and 3 (W2 and W3) in December 2016 and 2017, respectively.

Second, the research team designed the study with the aim of increasing dosage of Saleema (to the activities noted above) nationwide during the period between W2 and W3 of the campaign. This was done to create a ‘heavy-up’ effect, which is a media and advertising research methodology in which campaign implementation activities are increased over time by a known quantity in order to evaluate effects on an outcome [20]. The increase in campaign dose acts as a quasi-experimental control.

The strategy for the *Saleema* heavy-up design was to increase the media frequency level (measured by level of community activity in 4 intervention activities) by 100 percent during the period between W2 and W3 data collection time points (T2 and T3). Previous research has found that commercial media studies typically use a 50–100 percent increase in media delivery (reach and frequency). In a systematic review, Lodish and colleagues (1995) reported an average increase of 85 percent for established brands over 141 studies analyzed [21].

### Sample

In collaboration with researchers at the University of Khartoum, investigators drew a nationally representative sample of all 18 states in Sudan. The sampling frame was all households by state, obtained from the 2008 National Population and Households Census in Sudan. The unit of analysis was the head of household, male and female. The sample was stratified by i) state, and ii) gender (male and female heads of household).

A stratified two-stage sampling design was used. In the first stage, the research team selected a sample of clusters (within states, popular administrative units, or PAU) using probability proportional to size selection without replacement to get a sample of clusters from each stratum. In the second stage, the team used systematic selection, with equal probability, of 26 households from each selected cluster.

### Statistical power

Power was calculated based on descriptive normative beliefs about FGM given two time points of interest: at baseline; and 2 years post-baseline to compare differences in that outcome between individuals with high versus low exposure to *Saleema* intervention and control conditions. Studies have shown that reductions in descriptive norms are associated with reductions in intentions and ultimately in health behavior [22–23].

We employed a two-stage cluster sample design. In the first stage, we drew a sample of clusters (primary administrative units, PAU, within states) using Proportional to Population Sampling (PPS) selection without replacement to get a sample of clusters within each stratum. The strata were the 18 states in Sudan and gender (equal proportions female and male heads of household, HH).

In the second stage, we systematically selected, with equal probability, 26 HHs from each selected cluster. A systematic random sample of clusters was selected within each stratum with probability proportional to size in terms of households. The minimum required cluster size was 150 HH (others were excluded). After the selection of clusters, household listing operation was carried out. The formula used for the calculation of the sample size was as follows: z2 * r * (1-r) * deff; n = (RME * r)2 * RR. Where: n = the required sample size, (number of HHs); z = the value in the normal distribution that gives level of confidence 95%, (z = 2); r = predicted value of indicator (in target/base population), (r = 0.5); deff = the design effect; RME = relative margin of error at 95% confidence (RME = 0.048); RR = response rate.

The investigators assumed 25% descriptive social norms for FGM abandonment at baseline (belief that abandonment is common in my community) at baseline, given recent population data [4]. We further assumed a 50% recruitment rate (i.e., success in enrolling participants) [4], 80% power (1-β) to detect a difference of 15% in descriptive norms between high and low *Saleema* exposure conditions, design effect of 2, a 1.5 standard deviation (SD) of descriptive norms, and a two-sided significance level of 0.05. Based on these assumptions, we needed a baseline sample of 199 baseline participants per state, or 199 x 18 states = 3,582 participants drawn from across Sudan.

### Measures and instruments

In order to independently measure exposure to Saleema activities, the research team developed an exogenous measure of community activity to be collected at the local level. Liu and colleagues (2016) define exogenous measures as those that are assessed independently of outcomes, and also refer to them as ecological or unobtrusive measures [24]. More specifically, five categories of exogenous media measures have been identified: “(1) content analysis; (2) media ratings–gross or targeted rating points; (3) advertising expenditures; (4) records of promotional information at retail point-of-sale locations, and (5) hybrid measures that integrate self-reported information with one of the above four measures” [24].

The lead author, in collaboration with the UNICEF staff, developed a *Saleema* Evaluation and Monitoring System (SEAMS). The lead author trained State Councils for Child Welfare (SCCW) staff from all 18 states in Sudan to implement SEAMS to capture ongoing implementation activity following an observational data collection protocol. The system was supervised and administered by the NCCW to ensure consistent implementation. Thus SEAMS provides a measure of *Saleema* exposure independent of self-report. It is a database in Microsoft Access that allows SCCW staff to capture major activities under each of the basic *Saleema* initiatives, Sufara, Colors, community dialogue, and Born *Saleema*. SCCW representatives in each state uploaded SEAMS data monthly during the data collection period to a central data registry for monitoring and evaluation purposes. The lead author supervised SEAMS implementation.

We developed a questionnaire with extensive input from NCCW and UNICEF to be administered via tablet computer by trained GRACe interviewers in communities across Sudan. The questionnaire includes measures of aided and unaided awareness, message receptivity, scales for subjective and descriptive social norms about FGM and related knowledge, attitudes, and beliefs, as well as socio-demographic information about community, family, and individuals. The questionnaire specifically asked about awareness of the *Saleema* messages, exposure to specific Saleema media executions including community-level activities. It included validation techniques to compare self-reported campaign awareness data to placement data from the mass media Surfara *Saleema* spots and community-level campaign components in order to develop reliable estimates of campaign exposure [25].

The following is a summary of measures based on the *Saleema* conceptual framework. All measures were adapted from existing, validated scales adapted from the literature by the lead author [9, 13, 15, 17]. All questions were asked as 4-point agreement scales with don’t know options except where noted.

- **Self-Efficacy:** Agreement with the following statements
  ○ I am able not to practice FGM despite social pressure
  ○ I am confident my family will abandon FGM in the future
- **Positive Expectancies:** Agreement with the following statements
  ○ Abandonment will benefit the respondent’s community
  ○ Abandonment will benefit the respondent’s family
- **FGM Intentions:** Likelihood of practicing FGM in future
  ○ I will not practice cutting in the future
  ○ Would someone else in your family practice cutting in the future? (Yes/no)
  ○ Who is that person (mother/father/grandmother/grandfather/other)?
  ○ I will advise other people in my community who have daughters not to practice cutting
  ○ I will advise my friends to sign a pledge not to practice cutting in the future
  ○ If I have the chance, I will bring a friend or family members to a future *Saleema* event in my community
- **Descriptive Norms:** Perceived prevalence of FGM practice
  ○ Most people in my community practice cutting
  ○ Most of my friends practice cutting
- **Subjective Norms:** Perceived acceptability of FGM practice
  ○ It is appropriate for families in my community to practice cutting
  ○ Sudanese society in general considers it appropriate to practice cutting

**Brand Equity:** Brand equity has been shown to mediate the relationship between health media messages and attitudes/beliefs/behavioral outcomes [15–17] and consists of 4 second-order constructs: Brand loyalty, leadership/popularity, brand personality, brand awareness. Following are the individual measures used for *Saleema*:

○ I’d like to help spread the word about Saleema
○ I’d defend Saleema if someone was putting it down
○ I’d wear a Saleema t-shirt
○ If I had the chance, I would promote Saleema to my friends
○ Saleema program or materials is there in my community when I need it
○ I pay more attention to Saleema when I see it in my community
○ Saleema is the best program for people like me
○ Saleema is becoming more popular with people like me
○ Saleema is meant for people like me
○ I get a lot from being involved with Saleema
○ Being involved with Saleema is really worth it
○ The idea of keeping girls Saleema is growing and spreading in my community
○ When you think of Saleema you think: Girls should stay Saleema; Girls who are Saleema are marriageable; People should abandon cutting; It is God’s intention that girls stay Saleema; People who abandon CUTTING are making the right choice
○ Would you say that people in the Saleema advertising are: Good; Clean; Healthy; Successful; Happy.

- **Structural Access to/use of Media**
  ○ Hours of Media Use (respondent provided a numerical estimate)
  ○ Media channels used (yes/no to use of TV, radio, internet, mobile, video games, print)
- **Exposure & Reactions to *Saleema* Messages and Materials (yes/no)**
  ○ Awareness of *Saleema* language
  ○ (Unaided) Awareness of campaign tagline
- **Recall (yes/no)**
  ○ Confirmed or independent awareness of: TV/radio, Outdoor/print, Website, community events

The four social norms items (descriptive and injunctive) were examined using confirmatory factor analysis. Results of this analysis (presented below) indicated that the norms items loaded onto a single factor, which was used in subsequent analysis. The norms factor is based on the ordinal score—each question is scored 0, 1, 2, or 3. So the Norms score is an average of all four scores, and is a number between 0 and 3. We did this to preserve all the information we could from the responses. So, a 1 unit change in the score indicates a mean 1-point change in scores across all four questions, for example. The social norms factor is expressed as a percentage agreement or strong agreement in the results.

### Data collection

This study received approval for human subjects research from the AUW Research Ethics Board and in concurrence with the UNICEF Procedure for Ethical Standards in Research in October 2015. GRACe implemented data collection following the sampling plan in 3 waves annually in December 2015, 2016, and 2017. Teams of experienced enumerators recruited by GRACe received two days of training prior to implementation. The teams travelled to each state in Sudan and implemented data collection in person. In some cases, the tablet computers were not used when respondents were uncomfortable with that methodology, and paper questionnaires were recorded and later entered into the final database provided to the lead researcher. The identical questionnaire was used in all cases, and was administered by the interviewer following the same protocol.

### Analysis

Data from the 18 states for all 3 study waves were appended into a single dataset. Questions related to beliefs around Saleema, exposure to advertising, message receptivity, and FGMC intentions and social norms were compared descriptively. Each set of constructs was assessed using confirmatory factor analysis (CFA) to investigate scale variables that might represent each construct, using factor loading and Chronbach’s alpha (>0.6) tests to assess factor strength, following the widely cited Comrey & Lee (1992) criteria [26].

We then examined Saleema event participation by state. We summed the number of event participants for each state, and created per-capita indicators of community, government, and media event participation in order to reflect the scale of activity by state population. We examined the distribution of this indicator and noted a right-skewness in event participation across states. We log-transformed this variable to use as a linear predictor in regression modeling.

We conducted regression modeling, examining association with logged, per-capita event attendance and improved knowledge, attitudes and behaviors (KABs) around Saleema for wave 3. Intra-cluster coefficients (to account for the cluster randomized sampling design) were examined for each key outcome variable, and were determined to be minimal (F-test was insignificant) at the PAU-level. We then used state-level fixed effects regression modeling to compare respondents at waves 2 and 3 and examine the effect of increases or decreases in higher event participation, through use of an interaction term, in FGMC beliefs, intentions, attitudes, and norms. R software (version 3.3.2) was used in all analyses [27].

## Results

The total sample size at W3 was 3,824, at W2 it was 3,724, and at W1 it was 3,720, or 11,268 in total. The total sample was 55% female, mean age was 38.3 years, mean age among women at first cut (when applicable) was 6.7 years, and mean age at marriage (when applicable) was 20.2 years.

Data from SEAMS revealed wide variation overall in total events and event attendance by state. North Darfur state has mean per capita attendance of over 41 persons per event, while Sinnar state had a mean of less than 1 person per event. The average increase in logged per capita attendance from wave 2 to wave 3 was 65%, with wide variation between states. While less than the original target of 100%, this increase falls within the recommended range for heavy-up studies from the Lodish (1995) evidence review [21].

Table 1 summarizes overall agreement with the social norms items, which represent the main outcome of interest for *Saleema*. The overall observed trend is a gradual reduction in pro-FGM social norms. All 4 measures and the overall norms factor were lower at W3 than at W1, and 3 of the 4 measures and the overall factor declined at each wave.

**Table 1:** FGM social norms agreement (lower scores desirable).

Overall agreement by Wave, % (SE)	Most people in your community practice CUTTING	Most of my friends practice CUTTING	It is appropriate for families in my community to practice CUTTING	Sudanese society in general considers it appropriate to practice CUTTING	Norms Factor (Score based on 4 measured variables to left)
1	65.9% (0.8%)	58% (0.9%)	35% (0.8%)	62.9% (0.9%)	1.59 (0.01)
2	56.4% (0.9%)	47.6% (0.9%)	24.3% (0.7%)	48.4% (0.9%)	1.43 (0.01)
3	48.5% (0.9%)	41.9% (0.9%)	26% (0.7%)	44.8% (0.9%)	1.4 (0.01)
All	56.8% (0.5%)	49.1% (0.5%)	28.4% (0.4%)	51.9% (0.5%)	1.47 (0.01)

Table 2 summarizes results of social norms confirmatory factor analysis (CFA). The table displays mean scores on the 4-point agreement scale, % who agreed (strongly agree or agree), the factor loading, and scale alpha. Given that the scale is based on previous Social Norms measures, CFA was appropriate and the overall scale alpha of 0.8274 is acceptable [26].

**Table 2:** FGM social norms.

	Mean Score (1–4), SE	% Agree, SE	Factor Loading	Alpha
Most people in your community practice CUTTING	2.62	0.56	0.7807	0.8274
	(0.08)	(0.04)		
Most of my friends practice CUTTING	2.50	0.48	0.8247	
	(0.08)	(0.05)		
It is appropriate for families in my community to practice CUTTING	2.14	0.25	0.6091	
	(0.07)	(0.03)		
Sudanese society in general considers it appropriate to practice CUTTING	2.52	0.50	0.6992	
	(0.08)	(0.05)		

Table 3 summarizes multivariate regressions estimating the odds of improved (reduced) anti-FGM social norms at W3 based on self-reported exposure to *Saleema* campaign activity. The independent variable was recall of any campaign advertising or event activity in the respondent’s community. Self-reported exposure was positively associated with an improvement in each individual social norm measure and with the overall FGM social norms factor (coeff. = -0.329, *p* < .001). Additionally, having greater than a high school (HS) education was also associated with an improved FGM social norms factor.

**Table 3:** Self-reported Saleema advertising exposure regressions at wave 3.

	Most people in your community practice CUTTING	Most of my friends practice CUTTING	It is appropriate for families in my community to practice CUTTING	Sudanese society in general considers it appropriate to practice CUTTING	Social Norms Factor (Score based on 4 measured variables to left)
Any Ad Exposure	0.570***	0.539***	0.508***	0.418***	-0.329***
(Ref = No exposure)	(0.366, 0.775)	(0.306, 0.772)	(0.242, 0.774)	(0.203, 0.633)	(-0.375, -0.284)
Gender	1.157	1.21	0.919	1.300**	0.035
ref = Female	(0.943, 1.371)	(0.968, 1.453)	(0.634, 1.204)	(1.080, 1.520)	(-0.013, 0.083)
Age	1	1.004	1.002	1.001	-0.001
Continuous	(0.992, 1.008)	(0.995, 1.013)	(0.992, 1.012)	(0.993, 1.010)	(-0.003, 0.001)
HS or Greater Education	0.825	0.608***	0.494***	0.799	-0.183***
ref = No Education	(0.531, 1.120)	(0.263, 0.954)	(0.088, 0.901)	(0.496, 1.102)	(-0.246, -0.121)
Marital Status	0.91	0.941	0.867	0.749**	-0.013
ref = Not Married	(0.651, 1.169)	(0.645, 1.238)	(0.535, 1.199)	(0.489, 1.008)	(-0.071, 0.045)

*p < .05

**p < .01

***p < .001

Table 4 shows results from the log transformed per capita event exposure (independent variable) and its relationship with social norms and other Saleema outcomes of interest. This is a logistic regression for the individual variables and an Ordinary Least Squares model for the (continuous) social norms factor.

**Table 4:** Regressions of events exposure on social norms waves 2–3.

Odds Ratios, 95% CI	Most people in your community practice CUTTING	Most of my friends practice CUTTING	It is appropriate for families in my community to practice CUTTING	Sudanese society in general considers it appropriate to practice CUTTING	Social Norms Factor (Score based on 4 measured variables to left)
Wave 3	1.206	1.957***	1.199	2.629***	0.207***
Change from Wave 2–3	(0.831, 1.581)	(1.544, 2.370)	(0.750, 1.649)	(2.209, 3.050)	(0.126, 0.289)
Lpcw	1.341***	1.278***	1.047	1.083	0.081***
Logged per capita community events	(1.230, 1.452)	(1.145, 1.411)	(0.899, 1.195)	(0.970, 1.196)	(0.061, 0.101)
Wave 3:lpcw	0.746***	0.649***	0.885	0.664***	-0.146***
Change in logged community per capita variable from wave 2–3	(0.570, 0.922)	(0.448, 0.850)	(0.673, 1.097)	(0.461, 0.867)	(-0.183, -0.109)
Gender (Male)	1.141	1.013	0.874	1.442***	0.041**
ref = Female	(0.978, 1.303)	(0.830, 1.197)	(0.657, 1.091)	(1.270, 1.614)	(0.006, 0.076)
Age	1.005*	1.007*	1.005	1.010***	0.002***
Continuous	(0.999, 1.011)	(1.000, 1.013)	(0.997, 1.012)	(1.004, 1.016)	(0.001, 0.003)
High School (HS) education or More	0.914	0.792*	0.614***	0.946	0.136***
Ref = less than HS	(0.692, 1.136)	(0.537, 1.046)	(0.311, 0.917)	(0.712, 1.180)	(0.090, 0.181)
Marital status	0.808**	0.847	0.803*	0.809**	-0.069***
ref = Not Married	(0.622, 0.994)	(0.636, 1.057)	(0.562, 1.044)	(0.611, 1.006)	(-0.109, -0.028)

*p < .05

**p < .01

***p < .001

There is a strong and positive relationship between log transformed per capita event exposure measure and the individual social norms variables–higher levels of event exposure in wave 3 vs. wave 2 predicts a greater improvement (reduction) in FGM social norms among respondents from wave 2 to wave 3. Specifically, Table 4 shows changes in these key outcomes:

- Most people in your community practice CUTTING (OR = .746, or more than 25% less likely to agree with this statement, *p* < .001)
- Most of my friends practice CUTTING (OR = .649, or more than 35% less likely to agree with this statement, *p* < .001)
- It is appropriate for families in my community to practice CUTTING (no effect, OR = .885, which is in positive direction but *p* > .05)
- Sudanese society in general considers it appropriate to practice CUTTING (OR = .664, or more than 34% less likely to agree with this statement, *p* < .001)

Additionally, the overall social norms factor (right hand column) reflected an overall dose-response improvement—a greater increase in event exposure between waves 2–3 predicts a greater improvement (reduction) in the social norms factor (coeff. = -0.146, *p* < .001). Being male, being older (continuous age measure), having greater than High School (HS) education, and not being married were also associated with reduced pro-FGM social norms.

## Discussion

The *Saleema* outcome evaluation sheds light on the nature FGM in Sudan and provides evidence for the initiative’s effectiveness in changing social norms. Overall, findings suggest that social norms are changing in Sudan over time and during the *Saleema* implementation period. Dosage of *Saleema* has been effectively measured using both exogenous (independent) and endogenous (self-report) measures. Exposure to the initiative is associated with reduced pro-FGM social norms and other hypothesized outcomes. These findings both help to answer the initial questions that guided this research, and suggest the need for future programmatic initiatives and research on how to change FGM social norms and eliminate the practice.

This study demonstrated that *Saleema’s* social marketing strategy is effective in reducing pro-FGM social norms. The acceptability of FGM may be reduced, and the practice eventually eliminated, by focusing on normative belief change. The use of social norms strategies has been an increasing focus of public health efforts in many domains, including women’s health, gender equality, and social and economic empowerment [28]. For example, the Gates Foundation has organized a social norms community to share emerging research and encourage collaboration and advance the state of project design, measurement, and build evidence in the field. Gates held a social norms convening meeting in 2018 that aimed to 1) reflect on recent advances in social norms theory and practice, 2) identify gaps in evidence and practice; and 3) determine when normative approaches are relevant and may be best employed for programs [29].

The *Saleema* program brand and identity were widely recognized, as reflected in the self-reported recall data and increases in event attendance. *Saleema* represents as a source of strength, social support for ending FGM, and progress for Sudanese society. Identification with the brand was the primary program strategy to reduce pro-FGM social norms. This study does not address the question of whether brand identification led to reductions in pro-FGM social norms, but previous research shows that brand identification mediates outcomes of social marketing campaigns [17, 30]. Future studies should examine the potential mediating effect of *Saleema* brand identification on pro-FGM social norms. Understanding the mechanisms leading to reductions in pro-FGM social norms may enable future programs to accelerate abandonment in contexts such as Sudan.

Additionally, *Saleema* used a strategy of promoting social dialogue and ongoing conversation about FGM in order to reduce pro-FGM social norms. Previous qualitative research on *Saleema* shows that the program’s communication efforts can promote such dialogue [10]. But more research is needed on the mechanisms underlying social dialogue. How does *Saleema* promote it, and what can be done to amplify the conversation and bring in new voices? Future research should investigate whether other channels, such as digital media, can enhance community-based programs to abandon FGM.

Finally, with respect to measurement, the SEAMS proved to be an effective monitoring system that has promise for other social marketing campaigns in low and middle income (LMIC) country settings. Evaluation studies of health-related mass media campaigns have employed exposure to the campaign as the primary independent variable to predict behavioral outcomes [31]. However, exposure measures which lack reliability or validity can lead to either underestimating or failing to detect campaign effects [32–33]. The success of SEAMS hold significant promise for use in exogenous measurement of social marketing campaigns using a field-based monitoring system administered by local government or development officials in low resource settings.

This study has some limitations, mainly in that it is not a randomized controlled trial and the sample was not followed longitudinally. The heavy-up methodology was largely successful in that a substantial increase in exposure to *Saleema* (some 65% increase in per capita event attendance in 2017 compared to 2016), but this was less than the overall target. Nonetheless, results are robust and the quasi-experimental design is well supported in the research literature [34]. Results are consistent with other studies on FGM social norms [35].

## Conclusion

There is evidence that higher levels of exposure to *Saleema* leads to reduced pro-FGM social norms. The campaign has been effective in affecting its primary outcome of interest, improved social norms, and represents a promising strategy for abandonment of FGM in Sudan. This study suggests future FGM elimination programs in other LMIC may also benefit from adopting a social norms approach based on branding for behavior change. This study contributes to the growing literature on social norms.

## Supporting information

S1 File Further information on the nationally representative sampling procedure used in the Saleema study. (ZIP)
